# Thermal analysis during partial carbonizing process of rhubarb, moutan and burnet

**DOI:** 10.1371/journal.pone.0173946

**Published:** 2017-03-21

**Authors:** Junnan Ma, Xianglong Meng, Xiaohui Guo, Yaru Lan, Shuosheng Zhang

**Affiliations:** 1 Department of Herbology, College of Oriental Medicine, Dongguk University, Gyeongju, Republic of Korea; 2 Institute of Pharmaceutical and Food engineering, Shanxi University of Traditional Chinese Medicine, Shanxi, Jinzhong, P.R. China; 3 Key Laboratory of Carbon Materials, Institute of Coal Chemistry, Chinese Academy of Sciences, Shanxi, Taiyuan, China; University of Akron, UNITED STATES

## Abstract

Control of temperature and duration of partial carbonization for Chinese medicines have been mainly based on experience of the processors. No quantitative methods and parameters are available that can be used to precisely control the temperature and determine the energy changes during the process. In our research, with a simulated atmosphere air condition, the partial carbonization processes of three Chinese herb medicines rhubarb, moutan and burnet were simulated at different heating rates (5, 10 and 20°C • min^-1^) and analyzed by thermal gravimetric analysis (TGA) to quantify the upper limits of the temperature. The activation energy was calculated with Friedman, Kissinger-Akahira-Sunose (KAS) method and Ozawa-Flynn-Wall (OFW) methods in iso-conversional models and independent parallel reaction model (IPR). The upper temperatures were calculated to be 280, 184 and 246°C for rhubarb, moutan and burnet, respectively, at corresponding conversion rates of 0.4, 0.2 and 0.1. Calculation of the activation energy has been found impossible with the IPR model. Results obtained from the three iso-conversional methods were different. For rhubarb and burnet, the conversion rates at the upper temperature limits were at the highest or second highest activation energy, while for moutan, it was at the lowest value of activation energy. These results confirmed scientific rationales of traditional Chinese medicine theory that rhubarb and burnet be prepared at high temperature and moutan be prepared at medium temperature. Application of thermal analysis techniques would broaden and deepen traditional Chinese medicine research, and are applicable to the processing of medicinal materials including traditional Chinese medicines. Results obtained from the study could provide new ideas and methods for research to modernize the preparation of traditional Chinese medicines.

## Introduction

The processing of Chinese medicine is a traditional and unique pharmaceutical technology. It is based on Chinese medicine theory and is prepared according to syndrome, properties of the medicinals, and different requirements for prescriptions and preparations in accordance with the dialectical theory [1]. Due to its long history and rich content, it reflects the simple dialectical thoughts of traditional Chinese culture and is one of the characteristics of Chinese medicines.

Partial carbonization is a commonly used method in the making of some traditional Chinese medicines, where herbs are fried or roasted to increase carbon and tannin contents or to generate new hemostatic compounds, so the preparations can be used as hemostatic or antidiarrheal drugs [2–7]. However, this method has uncertainty in the temperature and duration in the partial carbonization process. For example, the temperature is selected by experience as medium (190–220°C) or high (220–300°C). This makes it difficult to determine the degree of preservation of the herb’s properties. Although there are requirements for the coloration of the carbonized herb drugs in accordance with “China Pharmacopoeia" and provincial or municipal "regulations for processing of Chinese medicine”, these requirements are qualitative, not quantitative. Currently, studies on improvement of processing of the carbonized herb drugs are focused on determination of the temperature and time [8–12]. Little is known about the compositional changes and conversion during the process [13–18]. Furthermore, there has been few studies regarding the methods and parameters that can be used to quantify the temperature and energy change during the partial carbonization process.

Thermal Analysis [19] is a technique to measure the physical properties of materials as they change with temperature and time under a programmed heating process. It includes thermal gravimetry (TG), derivative thermogravimetry (DTG), differential thermal analysis (DTA), thermo-mechanical analysis (TMA), dynamic mechanical analysis (DMA) and combined uses of thermal gravimetry with mass spectrometry (MS), Fourier transform infrared spectroscopy (FTIR), or gas chromatography (GC). In recent years, these technologies have been gradually applied to traditional Chinese medicine researches, such as thermal analysis of the chemical compositions [20], decomposition [21], and identification of product origin [22] or relatives [23].

We have applied the techniques to modernize the processing of traditional Chinese medicine for a long time. Previously, we used TG/DTG methods to simulate the processing procedure to qualitatively demonstrate the overall compositional changes in the preparation of Zushima [24–25], and analyzed the roles that pyrolysis in inert Ar played in the preparation of gallic acid [21], rhubarb, moutan and burnet [26]. We calculated the energy dynamics during the stir-frying process based on data obtained from the thermal analysis techniques. These results greatly enhance our experimental and theoretical understanding of the processing of traditional Chinese medicine. On the other hand, traditional Chinese medicines have complicated chemical compositions [27–28] and contain cellulose, hemicellulose and lignin [29–31]. Pyrolysis of these components often generates complicated pyrolysis curve, making it different to calculate the activation energy (Ea) in the partial carbonization process by dynamics mathematical methods.

In this study, the partial carbonization processes for preparing rhubarb, moutan and burnet were simulated using thermal gravimetric analysis (TGA) under a simulated air condition at different heating rates (5, 10 and 20°C • min^-1^) to quantify the upper temperature limit. The activation energy was calculated using the Friedman, Kissinger-Akahira-Sunose (KAS) and Ozawa-Flynn-Wall (OFW) methods in iso-conversional models [32] and independent parallel reaction model (IPR) [33]. The changes of the activation energy were analyzed to preliminarily define its quantification methods and evaluation indicators. The results will provide new ideas and methods to modernize the processing of Chinese medicine.

## Materials and methods

### Materials

Rhubarb (RH) (20141001, Gansu Province, P.R. China), Cortex moutan (CO) (141202, Gansu Province, P.R. China),burnet (SA) (20140505,Anhui Province, P.R. China)were purchased from Beijing Tongrentang Drug Store (No.104, South of Xinjian Road, Yinze district, Taiyuan City, Shanxi Province, P.R.China), and verified by Professor S. Zhang to be the roots and rhizomes of *Rheum tanguticum* Maxim. ex Balf, the root bark of *Paeonia suffruticosa* Andr. And the roots and rhizomes of *Sanguisorba officinalis* L., respectively. The herbs were grinded and passed through a sieve of 40 meshes.

### Water and ethanol extract

Water (RH) and ethanol (CO, SA) extracts of the herbs were made according to Chinese Pharmacopoeia (2010 edition) [34] and labelled as RHWSE, COASE and SAASE, respectively.

### Thermal Gravimetric Analysis (TGA)

Simultaneous thermal analyzer (STA-409C, NETZSCH, and Germany) was used. The herb sample (30 ± 5 mg) was placed in a crucible under a constant flow of simulated air (60 ml min^-1^, N_2_: O_2_ = 4:1). The temperature of the crucible was increased at 5, 10 and 20°C min^-1^ (heating rate) from room temperature to 600°C. Experiments were repeated twice.

### Kinetic model

Since Bryan Higgins observed the thermal effect of different heating temperature on the chalk and lime in 1780 [35], many studies have been done on kinetics of thermal analysis, and rapid progress has been made with advance of modern sciences. All proposed kinetic models obey the Arrhenius equation [36–37], as follows:

$$k(T)=A\text{exp}(\frac{-Ea}{RT})$$ (1)

where, *T* is the absolute temperature (K), R is the gas constant, *k(T)* is the temperature-dependent reaction rate, *A* is the pre-exponential factor, *Ea* is the activation energy. The physical meaning of *Ea* and *A* can be explained by molecular collision theory; the activation energy is the energy threshold that the molecule has to overcome to form a reaction product; the frequency factor *A* is the collision frequency at which all molecules collide.

Combustion and pyrolysis process of the Chinese medicines is a typical gas-solid reaction. Based on the Arrhenius equation, its kinetic equation in non-isothermal combustion and pyrolysis process can be expressed as follows [38]:

$$\frac{d\alpha }{dT}=\frac{k(T)}{\beta }f(\alpha )=\frac{A}{\beta }\text{exp}(\frac{-Ea}{RT})f(\alpha )$$ (2)

where *α* is the conversion rate or extent of reaction, $\frac{d\alpha }{dT}$ is the conversion rate in non- isothermal conditions, *β* is the rate at which the temperature increases (heating rate), *f*(*α)* is the mechanism function based on the selection of reaction mechanism and model, *Ea*, *A* and *f*(*α)* are three factors that characterize the pyrolysis kinetics.

A number of models in thermal analysis kinetics have been derived from the formula (2), as a result of improvement and interpretation of the formula, as well as the development of modern physics. Each of them has advantages and disadvantages [39–40], but all of them are able to calculate the activation energy and estimation of reaction mechanism during the pyrolysis and combustion. The models used in current research as follows:

### Independent Parallel Reactions models (IPR)

According to the first dynamic model, the pyrolysis and combustion of the medicines can be decomposed into N independent parallel reactions, each representing the pyrolysis reaction of an individual component. DTG curves were fitted using the Gauss Amp formula for multiple-peaks by PeakFit software (v4.12) for the three herb medicines. The kinetic equations used are as follows [32, 41]:

$$\frac{dm}{dt}=\sum_{i}c_{i}\frac{da_{i}}{dt},i=1,2,3,N$$ (3)

$$\frac{da_{i}}{dt}=A_{i}\text{exp}(-E_{i}/RT)(1-a_{i})$$ (4)

where $\frac{dm}{dt}$ and $\frac{d\alpha_{i}}{dt}$ are the rates of mass loss in the total reaction and in an individual reaction, *A*_*i*_ is the pre-exponential factor, *i* is a specific component, *E*_*i*_ is the activation energy, *α*_*i*_ is the conversion rate, *R* is the gas constant, *c*_*i*_ is the contribution of an individual reaction to the total reaction, and *T* is the absolute temperature (K).

### Iso-conversional model

The model is an independent model that can calculate the activation energy at certain conversion rate. The model is established irrespective of the impact of the heating rate and independent reaction mechanism on pyrolysis and combustion, while emphasizing the first-order process from the raw materials to pyrolysis products [33]. The method can be divided into integral and differential methods according to the sources of data (in integral method, the data are from TG curves, and in differential methods, the data are from DTG curves).

The basic formula in integral method is derived in steps 5 to 8:

$$T=\beta t+T_{o}$$ (5)

dT=βdt (6)

$$\frac{d\alpha }{dt}=k(T)f(\alpha )$$ (7)

$$g(\alpha )=\int_{0}^{\alpha }\frac{d\alpha }{f(\alpha )}=\int_{0}^{T}\frac{A}{\beta }e^{\left(-\frac{E}{RT}\right)}dT=\frac{AE}{\beta R}\int_{X}^{\infty }u^{-2}e^{-u}du=\frac{AE}{\beta R}P(x)$$ (8)

Depending on the definition of *p*(*x*) by different mathematicians, this model can be divided into different dynamics methods.

If *p*(*x*) = *x*^−2^*e*^−*x*^, then the model is defined as

$$\text{ln}(\frac{\beta }{T^{2}})=\text{ln}(\frac{AE}{Rg(\alpha )})-\frac{E}{RT},$$ (9)

namely, the Kissinger-Akahira-Sunose (KAS) method [42–43]; Similarly, if *p*(*x*) ≈ 10^−2.315+0.457*x*^, then the model is defined as

$$\text{log}\beta =\text{log}(\frac{AE}{Rg(\alpha )})-2.315-0.457\frac{E}{RT},$$ (10)

the Ozawa-Flynn-Wall (OFW) method [44,37].

The basic formula in differential method is derived as follows:

$$\frac{d\alpha }{dt}=\beta (\frac{d\alpha }{dT})=A\text{exp}(\frac{-E_{a}}{RT})f(\alpha )$$ (11)

Taking logarithm on both sides of eq (11) results in

$$\text{ln}\frac{d\alpha }{dt}=\text{ln}f(\alpha )+\text{ln}A-\frac{E}{RT},$$ (12)

the Friedman method [45].

*Ea* at a specific conversion rate can be calculated based on the linear relationships between the eqs (9) ($\text{ln}(\frac{\beta }{T^{2}})vs\frac{1}{T}$), (10) ($\text{log}\beta vs\frac{1}{T}$) and (11) ($\text{ln}\frac{d\alpha }{dt}vs\frac{1}{T}$).

## Results and discussion

### Determination of the upper temperature limit in the partial carbonization process based on the pyrolysis characteristics of the herbs and their extracts

In thermal analysis experiments, the air compositions are often changed to analyze the nature of physical and chemical changes in the thermal effect of the thermal analysis curves [46]. In early studies, we analyzed the relationship between the pyrolysis characteristics and the partial carbonization in the three medicines under inert gas of Ar [26]. However, under the inert air condition, what observed was the pyrolysis of the herbs themselves, which might be different from what happens in actual carbonization process.

**Fig 1:**
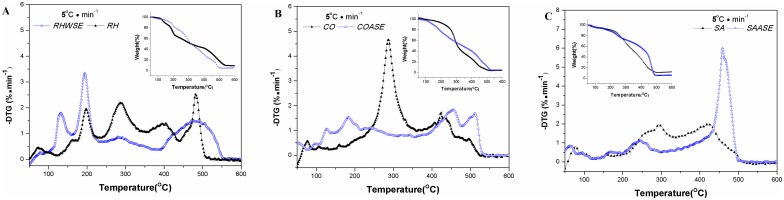
Combustion TG/DTG curves for medicinal herbs and extracts. The combustion characteristic for medicinal herbs and extracts sample ((A) RH and RHWSE, (B) CO and COASE, (C) SA and SAASE) were determined by thermal Analysis. They were placed in a crucible under a constant flow of simulated air (60 mL·min^-1^, N_2_: O_2_ = 4:1), and the temperature of the crucible was increased at 5°C·min^-1^ (heating rate) from room temperature to 600°C.

In this experiment, simulated atmosphere air was used to study the pyrolysis of the three medicines and their extract, and the results are shown in Fig 1. The results were evaluated using the post-carbonization property preservation of the medicinal ingredients in the extract as indicators. The temperatures at which the extracts reached the maximal rate of mass loss during the tradition carbonization temperature were defined as the upper limits. For rhubarb and moutan, they were 280 and 184°C. For burnet, it was 246°C. These data quantified the temperatures and confirmed the scientific rationale in traditional Chinese medicine that carbonization of rhubarb and burnet be made at high heat (220–300°C), and moutan at medium heat (190–200°C). The pyrolysis and combustion characteristics of the medicines and their extract are shown in Table 1.

**Table 1:** Charateristic parameters obtained from TG/DTG burning profiles of the *RH*, *CO and SA*.

Sample	β	T_v_	T_m_	T_f_	DTG_max_	Volatiles	Char
		(°C)	(°C)	(°C)	(%·min^-1^)	(%)	(%)
***RH***	5	122	286	438	2.19	74.06	19.49
	10	131	295	451	4.21	69.41	20.14
	20	138	305	468	8.21	66.06	24.17
***RHWSE***	5	100	193	380	3.33	45.01	34.52
***CO***	5	123	286	468	4.66	89.33	5.85
	10	127	297	493	9.11	84.4	5.91
	20	136	309	524	20.07	84.69	6.25
***COASE***	5	106	184	475	1.52	79.1	14.39
***SA***	5	130	292	450	1.99	76.43	5.95
	10	143	303	480	3.65	79.37	7.01
	20	151	314	500	6.92	74.88	12.16
***SAASE***	5	125	246	300	1.12	21.78	65.59

T_v_ = onset temperatures for volatile release and weight loss.

T_m_ = temperatures of maximum weight loss rate.

T_f_ = final combustion temperature detected as weight stabilization.

DTG_max_ = maximum weight loss rate.

Volatiles = weight of loss.

Char = weight of stabilization.

### Effect of heating rates on pyrolysis of Rhubarb, Burnet and Moutan

**Fig 2:**
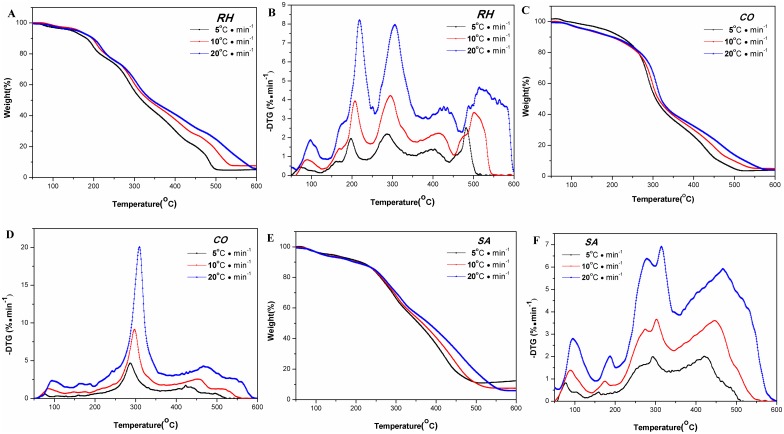
Combustion TG/DTG curves for the *RH*, *CO and SA* at differentβ. The combustion characteristics for medicinal herbs ((A, B) RH, (C, D) CO, (E, F) SA) were determined by thermal Analysis. They were placed in a crucible under a constant flow of simulated air (60 mL·min^-1^, N_2_: O_2_ = 4:1), and the temperature of the crucible was increased at 5,10 and 20°C·min^-1^ (heating rate) from room temperature to 600°C, respectively.

TG-DTG curves of pyrolysis and combustion of the medicines at different heating rates are shown in Fig 2. The curves became increasingly specific as the heating rate increased. For example, as the heating rate increased, the corresponding eigenvalues became greater. These eigenvalues included the pyrolysis starting temperature, the range of volatile-releasing temperature, the temperature at which the rates of mass loss peaks, and the temperature at which non-volatiles and fixed carbon stop burning (Table 1). At the same temperature, it was found that the lower the heating rate, the higher the burning completion, the more volatiles released, and the less the residue remained. This was because that at different heating rates, the heat transfer from outside to inside occurred at different speed. At higher heating rate, there would be greater thermal hysteresis between the furnace and the samples, leading to limited heat and mass transfer, and inadequate combustion. Conversely, at lower heating rate, the samples would have sufficient time to absorb heat for better pyrolysis. This might also be the reason why the peaks in DTG mass loss curve, which were poorly separated, became well separated at higher heating rate.

### Feasibility analysis to use IPR for determination of the activation energy in the pyrolysis and combustion process of the medicines

**Fig 3:**
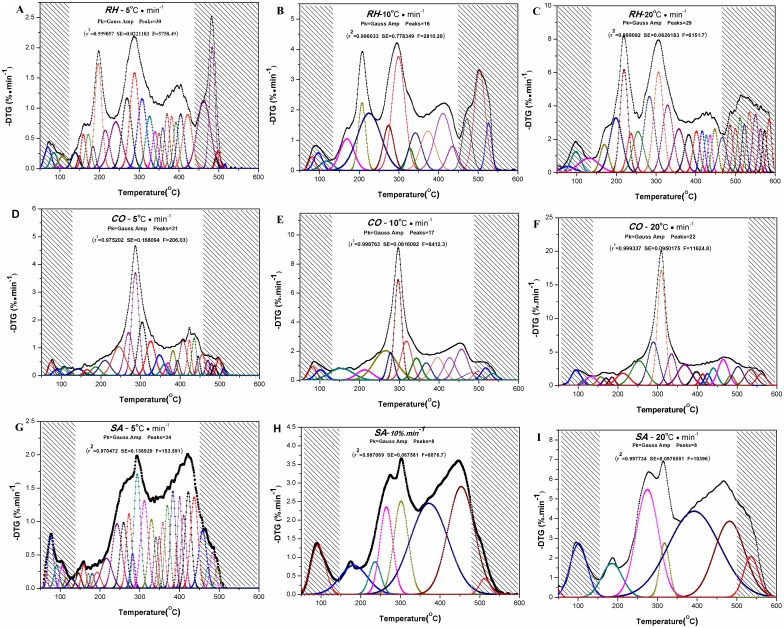
Comparison between experimental and calculated DTG curves using the IPR model, of *RH*, *CO and SA* at different heating rates. Multiple-peak fittings((A,B and C) RH, (D,E and F) CO, (G, H and I)were made according to the Gauss Amp equation using PeakFit (v4.12) at r2 ≥ 0.97 for DTG curves at different heating rates(5,10 and 20°C·min^-1^), respectively. And the non-shaded area is the interval of volatile.

Like other biomaterials, there are complicated and chained reactions in the pyrolysis and combustion process of the medicines. Each step in the process has its own activation energy [22]. Use of non-isothermal heating may result in overlap in DTG curves, namely, a peak in the curves is resulted from overlapping of the peaks from those of many chemical components that are present in the medicines. Multiple-peak fittings were made according to the Gauss Amp equation using PeakFit (v4.12) at r^2^ ≥ 0.97 for DTG curves at different heating rates. The peak fitting results and parameters for the three medicines are shown in Fig 3 and Table 2, and can be summarized as follows:

**Table 2:** Charateristic parameters obtained from calculated DTG curves using the IPR model, of *RH*, *CO and SA* at different heating rates.

Sample	*No*.	5%·min^-1^		10%·min^-1^		20%·min^-1^	
		Center	Percentage	Center	Percentage (%)	Center	Percentage (%)
		(°C)	(%)	(°C)		(°C)	
***RH***	reaction 1	31.95	0.05	80.82	1.02	43.56	0.49
	reaction 2	69.77	1.62	96.33	1.77	77.01	0.86
	reaction 3	84.66	1.16	117.58	1.35	97.68	2.03
	reaction 4	99.77	0.12	168.94	5.06	129.53	2.85
	reaction 5	107.62	1.21	206.89	5.57	169.54	2.33
	reaction 6	138.46	1.29	225.09	15.21	198.77	6.70
	reaction 7	148.85	0.52	273.92	5.27	219.21	7.90
	reaction 8	159.21	1.87	299.00	16.37	236.33	2.95
	reaction 9	171.64	2.06	328.13	1.80	254.63	4.45
	reaction 10	183.60	1.18	341.84	5.45	283.10	7.94
	reaction 11	196.81	7.98	373.31	6.66	305.99	9.43
	reaction 12	214.70	3.52	411.12	10.47	329.09	7.08
	reaction 13	240.80	6.35	435.64	2.96	357.35	4.81
	reaction 14	269.02	7.28	471.64	5.35	382.25	3.27
	reaction 15	287.81	9.32	503.98	12.45	401.54	2.82
	reaction 16	307.03	6.96	526.29	3.24	416.02	2.13
	reaction 17	325.85	4.48	-	-	426.48	1.58
	reaction 18	339.78	2.32	-	-	435.97	1.64
	reaction 19	349.57	1.85	-	-	448.13	2.59
	reaction 20	359.09	2.17	-	-	466.75	2.80
	reaction 21	370.04	3.06	-	-	486.25	2.67
	reaction 22	381.71	2.83	-	-	499.86	2.39
	reaction 23	392.36	2.68	-	-	511.67	2.78
	reaction 24	404.12	4.06	-	-	522.63	2.36
	reaction 25	421.62	5.54	-	-	534.98	3.39
	reaction 26	462.22	9.78	-	-	550.43	3.41
	reaction 27	483.57	7.39	-	-	563.57	2.02
	reaction 28	492.79	0.39	-	-	573.43	1.83
	reaction 29	498.22	0.88	-	-	584.87	2.50
	reaction 30	516.37	0.09	-	-	-	-
***CO***	reaction 1	74.57	1.59	81.93	2.50	33.85	0.10
	reaction 2	87.45	0.92	101.49	2.50	94.62	3.64
	reaction 3	107.56	1.18	153.91	6.31	113.26	0.67
	reaction 4	121.35	0.28	212.86	3.80	131.65	2.28
	reaction 5	128.88	0.12	264.89	15.25	157.23	1.60
	reaction 6	141.88	1.79	278.37	4.49	169.88	1.10
	reaction 7	158.61	0.15	297.11	16.44	185.12	1.20
	reaction 8	165.07	0.99	317.94	9.56	212.60	3.92
	reaction 9	186.74	1.87	343.45	5.11	253.17	8.99
	reaction 10	210.22	3.94	368.03	3.94	289.27	11.27
	reaction 11	245.51	8.62	396.07	6.62	309.38	21.45
	reaction 12	269.89	8.19	426.53	6.01	335.01	6.88
	reaction 13	286.81	16.93	456.43	9.10	367.86	6.98
	reaction 14	304.12	10.12	482.14	2.60	397.76	2.87
	reaction 15	325.49	7.39	497.86	1.81	412.96	1.70
	reaction 16	348.16	4.14	517.78	2.59	425.43	1.77
	reaction 17	360.54	1.92	534.80	1.35	440.23	3.52
	reaction 18	369.36	1.44	-	-	465.10	6.49
	reaction 19	381.98	3.25	-	-	484.41	1.70
	reaction 20	393.47	1.50	-	-	502.59	4.83
	reaction 21	407.49	5.67	-	-	534.18	4.31
	reaction 22	423.14	3.42	-	-	561.38	2.72
	reaction 23	435.40	3.98	-	-	-	-
	reaction 24	445.56	1.59	-	-	-	-
	reaction 25	455.56	2.93	-	-	-	-
	reaction 26	469.67	1.49	-	-	-	-
	reaction 27	478.89	1.04	-	-	-	-
	reaction 28	486.58	0.92	-	-	-	-
	reaction 29	497.79	1.99	-	-	-	-
	reaction 30	509.29	0.47	-	-	-	-
	reaction 31	517.31	0.18	-	-	-	-
***SA***	reaction 1	64.96	0.52	92.22	6.53	41.65	0.79
	reaction 2	76.60	2.90	182.29	7.39	101.45	7.64
	reaction 3	91.51	1.29	236.22	3.45	185.63	5.82
	reaction 4	104.80	1.10	264.54	10.35	274.66	20.12
	reaction 5	115.16	0.44	301.78	12.39	318.16	4.36
	reaction 6	128.82	0.70	372.56	31.86	392.48	36.87
	reaction 7	146.14	0.86	453.37	26.48	481.89	19.30
	reaction 8	159.36	1.17	512.54	1.55	536.00	5.10
	reaction 9	171.11	0.48	-	-	-	-
	reaction 10	180.81	0.66	-	-	-	-
	reaction 11	193.13	1.17	-	-	-	-
	reaction 12	216.93	3.44	-	-	-	-
	reaction 13	243.16	6.42	-	-	-	-
	reaction 14	259.56	4.69	-	-	-	-
	reaction 15	272.95	4.39	-	-	-	-
	reaction 16	282.15	1.60	-	-	-	-
	reaction 17	293.70	7.93	-	-	-	-
	reaction 18	311.83	6.84	-	-	-	-
	reaction 19	329.85	4.55	-	-	-	-
	reaction 20	340.99	2.33	-	-	-	-
	reaction 21	349.33	2.11	-	-	-	-
	reaction 22	358.12	3.16	-	-	-	-
	reaction 23	369.83	4.40	-	-	-	-
	reaction 24	383.66	5.27	-	-	-	-
	reaction 25	392.52	1.38	-	-	-	-
	reaction 26	400.50	4.18	-	-	-	-
	reaction 27	410.59	3.75	-	-	-	-
	reaction 28	422.10	6.38	-	-	-	-
	reaction 29	437.22	7.12	-	-	-	-
	reaction 30	458.24	4.91	-	-	-	-
	reaction 31	471.23	0.89	-	-	-	-
	reaction 32	483.41	1.94	-	-	-	-
	reaction 33	495.97	1.01	-	-	-	-
	reaction 34	508.20	0.02	-	-	-	-

Center = Correspond temperature of DTGmax for reaction.

Percentage = the area of DTG curves for reaction account for total area.

1. When β = 5°C • min^-1^, the fitted peak of DTG curves for rhubarb was located at 287.81°C in the reaction 15, moutan at 186.74°C in the reaction 9 and burnet at 243.16°C in the reaction 13. The findings further confirmed the conclusions obtained in section 3.1 that it is scientifically rational to use the post-carbonization property preservation of the medicinal ingredients in the extracts to determine the upper temperature limits in the partial carbonization processes of the three medicines.
2. Since the three medicines are made from herbs, there were mass loss peaks associated with biomass in the fitted peaks in the DTG curves. For example, the thermal mass loss peaks of hemicellulose, cellulose and lignin can be found in the DTG curves of the three medicines at different heating temperature at 300, 380 and 420°C, respectively [47–53]. Therefore, when using (TGA) for the medicine carbonization research, the medicinal components and pyrolysis characteristics of biomass should be taken into consideration.
3. Heating rate β was found to have greater impact on the outcomes of the peak fitting in DTG curves for the medicines. When β was lower (5°C • min^-1^) or higher (20°C • min^-1^), there were more independent reactions in the peak fitting than when β was at medium level (10°C • min^-1^). This might be because at lower heating rate, the ingredients in the medicines have sufficient time to combust and pyrolyze, resulting in overlapped DTG curves due to presence of large number of independent reactions; while at higher heating rate, the independent reactions were incomplete, leading to the overlapped DTG curves. Therefore, when use TG for medicines carbonization study, heating rate β should be about 10°C • min^-1^.
4. In the field of biomaterial research, only three components hemicellulose, cellulose and lignin are generally taken into consideration for pyrolysis kinetics studies with IPR models [49–50]. Therefore, their DTG curves are fitted as three independent and parallel reactions. However, in this experiment, the number of the independent reactions derived from the peak fitting of the DTG curves at different heating temperature for the three medicines were between 8 and 34, much more than that that of the biomaterials in independent parallel reactions [49–50].
5. With reference to formulas (3)–(4), $\frac{d\alpha_{i}}{dt}$ may be obtained from the DTG curves fitted from the peaks of the independent parallel reactions, *c*_*i*_ can be approximated by the percentage of the total peak area that is between DTG curves and the baseline for a specific independent reaction over the total peak area that is between DTG curves and the baseline for all reactions. *α*_*i*_ is still not possible to be resolved for a specific independent reaction.

In summary, due to complexity of the chemical composition in the medicines and the differences between multiple-peak fitting and real reaction, it is still difficult to use IPR model for calculating the activation energy in the pyrolysis and combustion processes for traditional Chinese medicine. In this article, we only analyzed the feasibility to use IPR model for the calculation of the activation energy in the pyrolysis and combustion process. No in-depth calculation was attempted.

### Determination of the activation energy in the medicinal pyrolysis and combustion process using iso-conversional models

**Fig 4:**
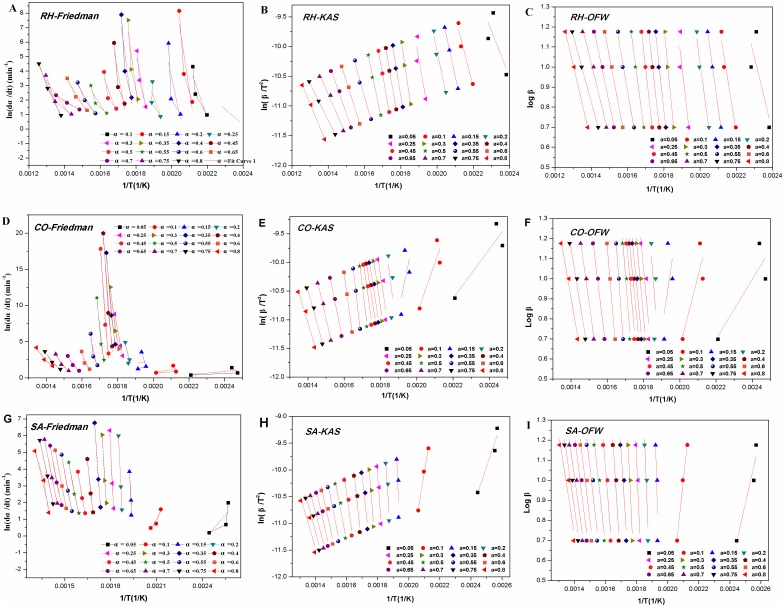
Estimation of activation energy using the Friedman, KAS and OFW models for *RH*, *CO and SA*. Linear relationship curves ((A, B and C) RH, (D, E and F) CO, (G, H and I) were made according to Friedman, KAS and OFW models at different conversion rates (from 0.05 to 0.8), respectively.

**Fig 5:**
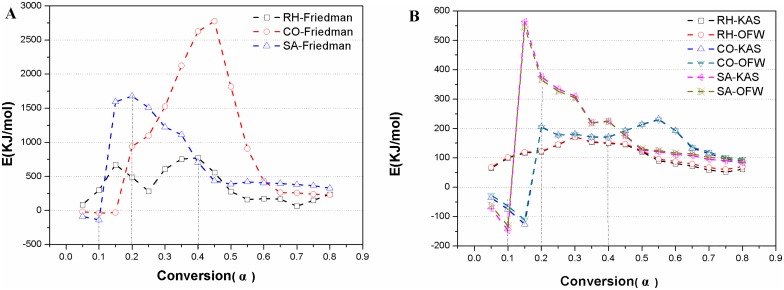
Tendency of activation energy using the Friedman, KAS and OFW models for *RH*, *CO and SA*. Fig A shows the tendency of activation energy using the Friedman model for *RH*, *CO and SA*, Fig B shows the tendency of activation energy using the KAS and OFW models for *RH*, *CO and SA*.

Compared to IPR model, a constant α and reaction rate constant *k* are assumed to be dependent on reaction temperature (*T)* in iso-conversional model. Therefore, this method avoids the system errors of the Arrhenius parameter estimation and can be performed without knowing the reaction mechanism behind the combustion and pyrolysis process [33]. It is, therefore, easy and simple, and has been widely used. The linear relationships for the activation energy were calculated for the medicines using the KAS (eq 9), OFW (eq 10) and Friedman (eq 12) methods as discussed in section 2.4.2 for iso-conversional kinetics models (Fig 4). They can be summarized as follows:

1. The activation energy can be calculated based on the slopes of the linear relationships. The linear correlation coefficient (R^2^) was relative small at some conversion rates, particularly when the rate was less than 0.2; also, there were differences between the R^2^ derived from different equations. These would increase the experimental error. However, since there were many correlation coefficients with higher values, and the activation energy of a specific reaction was a constant, it was still possible to figure out the activation energy based on the linear relationships with poor correlation coefficients. This would allow us to have an overview of the activation energy dynamics [51].
2. As shown in Table 3, among the three methods used, the liner relationships for rhubarb based on the KAS and OFW methods were better, particularly when the conversion rate was greater than 0.25. However, for moutan and burnet, the Friedman method was better than the KAS and OFW methods, particularly when the conversion rate was more than 0.2. These data suggested that the activation energy for different medicines should be calculated with different methods. Furthermore, as shown in Table 4, for the three medicines, the averages of the activation energy calculated by the KAS and OFW methods were pretty close each other, but remarkably smaller than that derived from the Friedman method. These differences might be resulted from different data sources. In the integral methods, there are Picard iterations for the conversion rates [52], and successive approximation would be prone to error. On the other hand, the conversion rate derived from the differential method is not affected by integration of temperature, where all relevant dynamics parameters can be calculated directly. However, the experimental data are highly sensitive to noise, and could result in highly scattered DTG curves [53].
3. The conversion rates were approximately 0.4, 0.1 and 0.2 at the upper temperature limits of 280, 184, and 246°C, respectively, as determined in section 3.1 for the three medicines. The changes in the conversion rates and activation energy for the medicines are shown in Fig 5, where the curves derived from the KAS and OFW methods were similar. In three kinetic models, rhubarb and burnet reached the highest or second highest activation energy at the conversion rates of 0.4 and 0.2, respectively. Moutan reached the lowest point when the conversion rate was 0.1. Therefore, rhubarb and burnet are prepared with higher energy input, while moutan needs less energy. The findings further demonstrated the scientific rationales in the partial carbonization from the energy requirements that rhubarb and burnet need high temperature and moutan needs medium temperature.

**Table 3:** TGA pyrolysis of *RH*, *CO and SA* activation energies (Ea) and correlation factors (R^2^) for conversion values using the Friedman, KAS and OFW models.

Sample	Conversion(α)	Activation energy Friedman model (KJ/mol)	R^2^	Activation energy KAS model (KJ/mol)	R^2^	Activation energy OFW model (KJ/mol)	R^2^
***RH***	0.05	85.13	0.4316	64.28	0.1715	67.86	0.2706
	0.1	298.65	0.6464	99.57	0.9032	101.98	0.9157
	0.15	670.91	0.7196	116.83	0.9996	118.70	0.9996
	0.2	486.51	0.2854	120.43	0.9316	122.40	0.9400
	0.25	281.96	0.1591	144.31	0.5895	145.56	0.6261
	0.3	606.28	0.8325	171.58	0.9713	171.90	0.9739
	0.35	753.25	0.7293	153.37	0.9832	154.67	0.9850
	0.4	773.26	0.7024	149.56	0.9966	151.21	0.9968
	0.45	556.40	0.6936	145.12	1.0000	147.31	1.0000
	0.5	277.62	0.6238	120.81	0.9927	124.42	0.9937
	0.55	161.57	0.6998	90.16	0.9826	95.63	0.9854
	0.6	173.14	0.9218	80.81	0.9967	87.21	0.9975
	0.65	164.44	0.9390	72.19	0.9874	79.40	0.9912
	0.7	62.39	0.9892	58.34	0.9634	66.60	0.9757
	0.75	147.12	0.7562	51.70	1.0000	60.68	0.9999
	0.8	236.81	0.9826	61.41	0.9971	70.30	0.9983
***CO***	0.05	-19.41	-0.1791	-35.69	0.6911	-27.17	0.5580
	0.1	-37.11	-0.4696	-75.39	0.6176	-64.06	0.5430
	0.15	-32.44	-0.9903	-125.92	-0.2810	-111.53	-0.3444
	0.2	939.56	0.6293	205.41	-0.3813	203.75	-0.3444
	0.25	1097.16	0.9973	177.35	0.6604	177.36	0.6870
	0.3	1525.36	0.9538	181.04	0.8798	181.06	0.8908
	0.35	2122.20	0.9603	170.62	0.8953	171.07	0.9054
	0.4	2624.97	0.9476	170.30	0.8951	171.07	0.9054
	0.45	2774.20	0.9550	192.38	0.8604	192.15	0.8731
	0.5	1816.22	0.9291	213.93	0.8808	212.71	0.8903
	0.55	906.20	0.7341	231.39	0.9982	229.38	0.9983
	0.6	432.58	0.8272	192.93	0.9998	193.29	0.9998
	0.65	261.41	0.9145	132.81	0.9859	136.48	0.9879
	0.7	257.53	0.9107	114.60	0.9849	119.66	0.9876
	0.75	236.73	0.9534	95.01	0.9696	101.45	0.9760
	0.8	229.94	0.9282	88.63	0.9551	95.69	0.9659
***SA***	0.05	-88.61	0.2234	-71.99	0.8867	-62.13	0.8644
	0.1	-138.40	0.7593	-147.10	0.9831	-132.28	0.9810
	0.15	1593.91	0.8619	563.97	0.3515	545.00	0.3652
	0.2	1676.91	0.9999	375.82	0.7451	366.14	0.7558
	0.25	1507.60	0.9734	334.70	0.8828	327.00	0.8883
	0.3	1221.76	0.9757	310.26	0.8882	304.27	0.8940
	0.35	1110.52	0.9964	219.73	0.8101	218.28	0.8244
	0.4	704.91	0.9880	225.04	0.8756	223.56	0.8861
	0.45	432.71	0.9930	175.42	0.8676	176.68	0.8814
	0.5	385.29	0.9836	126.35	0.9046	130.20	0.9191
	0.55	420.49	0.9983	120.59	0.8226	125.02	0.8487
	0.6	402.89	1.0000	111.11	0.8196	116.38	0.8497
	0.65	396.47	0.9941	107.80	0.8690	113.51	0.8920
	0.7	379.67	0.9980	94.83	0.8668	101.42	0.8940
	0.75	362.42	0.9985	90.30	0.8450	97.37	0.8783
	0.8	327.05	0.9322	83.14	0.7639	90.87	0.8169

**Table 4:** Values of activation energy obtained by Friedman, KAS and OFW models.

Samples	Activation energy*(KJ/mol)		
	Friedman model	KAS model	OFW model
*RH*	358.47	106.28	110.36
*CO*	945.94	120.59	123.90
*SA*	668.47	170.00	171.33

* The activation energy was calculated as arithmetic average of the several E values obtained for the different Conversion(α) show in Table 3.

Although the temperature requirements for the medicine preparation during the partial carbonization processes can be well explained based on the conversion rates calculated from the iso-conversional kinetic model, precautions should be taken on the applicability of the different models to avoid large errors.

## Conclusions

The partial carbonization temperatures were qualitatively determined for rhubarb, burnet and moutan prepared at higher or medium temperature in traditional Chinese medicine process theory. We analyzed the pyrolysis and combustion parameters from kinetics perspectives and discussed the feasibility to modernize the research on the partial carbonization in the frame works of kinetic models. The activation energy, as well as the relationship between the conversion rate and the activation energy, has been calculated using various kinetic models. The scientific rationales that rhubarb and burnet be made at high temperature and moutan at medium temperature as instructed in the traditional Chinese medicine process theory have been confirmed. Our study shows that thermal analysis techniques can be applied to broaden and deepen the research on traditional Chinese medicine, and are applicable to disciplines of other medicine preparation in the processing of Chinese medicines. These applications would provide new ideas and methods for current research on the Chinese medicine preparation.

## Supporting information

S1 Fig Combustion TG/DTG curves for medicinal herbs and extracts. (XLSX)

S2 Fig Combustion TG/DTG curves for the RH, MO and SA at differentβ. (XLSX)

S3 Fig Comparison between experimental and calculated DTG curves using the IPR model, of RH, CO and SA at different heating rates. (XLSX)

S4 Fig Estimation of activation energy using the Friedman, KAS and OFW models for RH, CO and SA. (XLSX)

S5 Fig Tendency of activation energy using the Friedman, KAS and OFW models for RH, CO and SA. (XLSX)
